# Therapeutic effects and mechanisms of Xinmaitong formula for type 2 diabetes mellitus via GLP-1R signaling

**DOI:** 10.3389/fphar.2025.1575450

**Published:** 2025-04-09

**Authors:** Weidong Pu, Yang Pan, Kang Yang, Jian Gao, Fen Tian, Jingrui Song, Yubing Huang, Yanmei Li

**Affiliations:** ^1^ State Key Laboratory of Discovery and Utilization of Functional Components in Traditional Chinese Medicine, Guizhou Medical University, Guiyang, China; ^2^ Natural Products Research Center of Guizhou Province, Guizhou Medical University, Guiyang, China; ^3^ School of Pharmaceutical Sciences, Guizhou Medical University, University Town, Guiyang, Guizhou, China

**Keywords:** Xinmaitong formula, GLP-1R, T2DM, cAMP, Ca^2+^

## Abstract

**Introduction:**

Traditional Chinese Medicine (TCM) theory posits that type 2 diabetes mellitus (T2DM) characterized by Qi and Yin deficiency, is associated with elevated blood lipid levels. The Xinmaitong formula (XMT) is a folk remedy believed to lower blood lipid levels. However, the functional components and molecular mechanisms through which XMT exerts its anti-diabetic effects remain to be elucidated. This study aimed to investigate the therapeutic effects and potential mechanisms of XMT in the treatment of T2DM, focusing on the glucagon-like peptide-1 receptor (GLP-1R) signaling pathway.

**Methods:**

A TCM formula that promotes GLP-1R expression was screened using a GLP-1R promoter-dependent luciferase reporter gene vector (PGL3-GLP-1R-luc). The T2DM mouse model was established using a high-fat diet and streptozotocin (STZ). Blood glucose levels were measured using a glucometer and oral glucose tolerance test (OGTT). Serum biochemical parameters and insulin levels were also assessed. Organ pathology in mice was evaluated using hematoxylin and eosin (H&E) staining. Immunofluorescence (IF) was employed to observe changes in insulin and GLP-1R expression in the pancreas of mice. The effects of medicated serum on Min6 cell growth were examined using a methyl thiazolyl tetrazolium (MTT) assay. A Min6 cell injury model was established to detect cAMP and Ca^2+^ concentrations. Ultra high-performance liquid chromatography-mass spectrometry (UHPLC-MS) was used to identify blood-absorbed components of XMT.

**Results:**

Luciferase reporter constructs driven by GLP-1R promoter response elements analysis identified that TCM formula XMT promoted GLP-1R expression. *In vivo* experiments demonstrated that XMT significantly reduced fasting blood glucose levels in T2DM mice and improved OGTT results. It also exhibited protective effects on islet tissues, notably increasing GLP-1R expression and insulin secretion in the pancreas. Biochemical markers indicated no significant adverse effects on liver or kidney function following XMT administration. After treatment with palmitic acid (PA), GLP-1R expression in Min6 cells was significantly decreased. However, treatment with XMT upregulated GLP-1R expression. Additionally, cyclic adenosine monophosphate (cAMP) and Ca^2+^ exhibited substantial improvements, and the key pancreatic growth protein PDX1 was activated.

**Conclusion:**

XMT exerts hypoglycemic effects by upregulating GLP-1R gene expression, enhancing GLP-1R protein synthesis, and subsequently promoting cAMP release. This process activates Ca^2+^ influx in pancreatic β-cells, triggering insulin exocytosis from islet cells.

## 1 Introduction

Type 2 diabetes mellitus (T2DM) is a metabolic disease characterized by persistent hyperglycemia (Mosili et al., 2024), typically resulting from insufficient insulin secretion or insulin resistance (IR) (Balakumar et al., 2023). The rapid progression of diabetes poses significant challenges to human health (Cloete, 2022). According to the latest data from the International Diabetes Federation (IDF) in 2021, the number of diabetes patients is projected to reach 783 million by 2045 (Sun et al., 2022), with T2DM accounting for over 90% of cases (Zheng et al., 2018). In recent years, the prevalence and mortality rates associated with hyperglycemia-induced complications have continued to increase (Dai et al., 2016), making the health issues caused by T2DM a focal point in developing drugs for metabolic diseases.

Currently, treatments for T2DM include surgical interventions (Sandoval and Patti, 2023), dietary control (Chen et al., 2023), and exercise therapy (Magkos et al., 2020), with pharmacological therapy remaining the primary approach (Blonde et al., 2018). Antidiabetic drugs widely used in clinical practice include insulin preparations (Horlyck-Romanovsky and Sumner, 2019), insulin secretagogues (Su et al., 2023), and insulin sensitizers (Lim et al., 2024). However, these treatments are often associated with side effects such as hypoglycemia, gastrointestinal discomfort, and lactic acidosis (Fei et al., 2024). Moreover, these drugs do not protect damaged pancreatic β-cells, as they focus solely on insulin and its targets while neglecting the functional preservation of β-cells.

Extensive research has demonstrated that in pancreatic β-cells, the GLP-1 receptor (GLP-1R) promotes cell proliferation, inhibits apoptosis, and enhances insulin secretion (Zhao et al., 2021). GLP-1R provides significant protection to pancreatic function, when activated by GLP-1, releases cAMP as a second messenger to activate downstream signaling pathways, thereby exerting antidiabetic effects (Wan et al., 2023). Thus, GLP-1R is emerging as a promising therapeutic target for T2DM and holds considerable research significance.

Traditional Chinese Medicine (TCM) is often regarded as a “brilliant jewel” in the global medical field (Ni et al., 2024). Characterized by its multi-component, multi-target, and holistic approach to regulation (Hao et al., 2024), TCM has been widely used in China for thousands of years, showing significant potential in improving health and preventing or treating various diseases. Additionally, TCM generally has relatively mild adverse effects when used in patients.

Recent studies have highlighted the potential of TCM in managing T2DM. For example, Huanglian Decoction (HLD) has been shown to regulate glucose metabolism disorders in T2DM rats (Pan et al., 2020). Similarly, the Jiang-Tang-San-Huang (JTSH) pill significantly improves hyperglycemia, insulin resistance (IR), hyperlipidemia, and pathological changes in the pancreas, liver, kidneys, and intestines of T2DM rats (Tawulie et al., 2023). Xiehuo-Guzheng granules alleviate β-cell dedifferentiation by regulating intestinal flora and its metabolites in T2DM (Cao et al., 2024). In addition, TCM also has a significant effect on the improvement of T2DM complications. It has been reported that Liuhuang decoction (LHD) also has the potential efficacy to improve endothelial dysfunction (Xu et al., 2025). These findings underscore the promising potential of TCM formulas and suggest further in-depth exploration in the treatment of T2DM.

XMT, a traditional folk medicine, comprises ten Chinese herbs, including *Angelicae Sinensis Radix, Cassiae Semen, Uncariae Ramulus Cum Uncis, Achyranthis Bidentatae Radix, Salviae Miltiorrhizae Radix et Rhizoma, Puerariae Thomsonii Radix, Flos Sophorae Immaturus, Ilex pubescens Hook. et Arn, Prunellae Spica, and Notoginseng Radix et Rhizoma.* XMT has been used in folk medicine to treat hypertension and hyperlipidemia, with functions such as promoting blood circulation, unblocking meridians, nourishing the heart, and lowering blood pressure and lipid levels. Among them, *Angelicae Sinensis Radix*, *Achyranthis Bidentatae Radix*, *Salviae Miltiorrhizae Radix et Rhizoma*, *I. pubescens Hook. et Arn* and *Notoginseng Radix et Rhizoma* belong to the category of tonifying blood and activating blood, with the core of harmonizing blood vessels and tonifying blood deficiency. *Cassiae Semen*, *Prunellae Spica*, *Uncariae Ramulus Cum Uncis* and *Flos Sophorae Immaturus* belong to the category of clearing heat and calming liver, with clearing liver heat, calming liver yang and cooling liver blood as the core. *Puerariae Thomsonii Radix* belongs to the category of relieving exterior and dredging collaterals, which is mainly to dispel wind evil. However, the effects of XMT on T2DM remain unexplored. In this study, we aim to investigate the hypoglycemic effects of XMT on T2DM and elucidate its underlying mechanisms.

## 2 Materials and methods

### 2.1 Drugs and reagents

XMT purchased from Guizhou Yibai Pharmaceutical Co., Ltd. (Guizhou, China). Metformin (MET) was purchased from Merck Pharmaceuticals (Nantong, China). Lipofectamine™ 2000 transfection reagent and PureLink™ HiPure plasmid extraction kits were obtained from Thermo Fisher (USA). Steady-Lumi™ II luciferase assay reagent was sourced from Beyotime (Shanghai, China). High-fat feed was purchased from Xietong Biotechnology (Jiangsu, China), and streptozotocin (STZ) was obtained from Sigma (USA). Kits for measuring HDL-C, LDL-C, TC, TG, GLU, AST, ALT, BUN, and CR were purchased from Jiancheng Bioengineering Institute (Nanjing, China). Ultra-pure water was prepared using a Millipore Alpha-Q water purification system (Millipore, USA). The calcium fluorescent probe Fluo-3 AM was purchased from Solarbio (Beijing, China). Antibodies for β-Actin were obtained from Proteintech (Wuhan, China); GLP-1R from Immunoway (USA); PDX-1 from Signalway Antibody (USA); and insulin from Servicebio (Wuhan, China). cAMP and insulin ELISA kits were obtained from Jianglai Biological (Shanghai, China).

### 2.2 Cell culture

HEK-293T and Min6 cells used in this study were kindly provided by Dr. Yaacov Ben-David (Guizhou Medical University). HEK-293T cells were cultured in DMEM medium (Gibco, USA) supplemented with 5% fetal bovine serum (FBS, Gibco, USA) at 37°C in a humidified incubator with 5% CO_2_. Min6 cells were cultured in RPMI 1640 medium (Gibco, USA) supplemented with 5% FBS (Gibco, USA) and 50 μmol/L β-mercaptoethanol (MCE, USA) at 37°C in 5% CO_2_. Both cell lines were maintained under standard cell culture conditions in a 37°C incubator with 5% CO_2_.

### 2.3 Preparation of medicated serum

The medicated serum was prepared as follows: Eighteen male SD rats (SPF grade, 6 weeks old, 200 ± 20 g) were purchased from Guizhou Huijiu Biological Technology Co., Ltd. (Guizhou, China). After a 7-day acclimatization period, the rats were randomly divided into blank group and medication groups. The dosage of clinical ethnomedicines was calculated based on the human-to-rat conversion ratio and multiplied by five times the clinical equivalent dose. The XMT dose was 2.268 g/kg. All medicines were dissolved in distilled water to the required concentration and administered by gavage at a volume of 1 mL/100 g body weight. Rats in the blank group received the same volume of distilled water. Administration was performed for five consecutive days. Food was withheld at 10:00 p.m. the night before the final administration, and the final dose was given at 8:00 a.m. the following morning. Approximately 1.5–2 h after the final administration, rats were anesthetized with isoflurane, and 10 mL of blood was collected from the abdominal aorta. Blood samples were allowed to clot at room temperature for 2 h and centrifuged at 4°C for 15 min at 3,000 rpm. The supernatant was heat-inactivated in a 56°C water bath for 30 min, filtered under aseptic conditions to remove bacteria, aliquoted, and stored at −20°C for later use.

### 2.4 Construction of PGL-3-GLP-1R-luc plasmid and drug screening

The PGL-3-GLP-1R-luc plasmid was constructed by cloning the GLP-1R gene promoter into the upstream region of the PGL-3-Basic luciferase reporter vector. This plasmid was transfected into HEK-293T cells using Lipofectamine™ 2000, and drugs that enhance GLP-1R expression were screened using a luciferase reporter assay kit.

Transfection Protocol: Cell Preparation: One day prior to transfection, HEK-293T cells were seeded in a 6-well plate to ensure 70%–80% confluence at the time of transfection. On the day of transfection, cells were observed microscopically, and the medium was replaced with serum-free DMEM.

Reagent Preparation: Tube 1: Add 150 µL of serum-free DMEM and 8 µL of Lipofectamine™ 2000, mix gently, and incubate for 5 min. Tube 2: Add 200 µL of serum-free DMEM and 4 µg of plasmid DNA, mix gently, and incubate for 5 min. Combine the contents of both tubes, gently mix, and incubate for 20 min to form Lipofectamine-DNA complexes.

Transfection: Add 250 µL of the Lipofectamine-DNA mixture dropwise to each well in the 6-well plate and incubate for 8 h. Afterward, replace the medium with DMEM containing 5% FBS and incubate for an additional 12 h.

Drug Screening: Transfected HEK-293T cells were seeded in 96-well plates at a density of 10 × 10^3^ cells per well in 80 µL of DMEM medium. After 6–8 h of adhesion, drug-containing serum was added at concentrations of 5%, 10%, 15%, and 20%. Blank wells were adjusted to 100 µL with serum-free DMEM. After 24 h of treatment, add 100 µL of Steady-Lumi™ II firefly luciferase assay reagent to each well under dark conditions. Allow the reaction to proceed for 5–10 min, then transfer the mixture to a 96-well white plate and measure luminescence using a microplate reader. Relative luciferase activity was calculated for analysis.

### 2.5 Validation of XMT’s promotion of GLP-1R expression in Min6 cells

mRNA Expression Analysis: Min6 cells were treated with XMT for 24 h, and total RNA was extracted using Trizol reagent (Invitrogen, USA). cDNA synthesis was performed using a reverse transcription kit (TaKaRa, Dalian, China). Real-time quantitative PCR was conducted with SYBR Green premix, following the manufacturer’s protocol. Relative mRNA levels were normalized and calculated using the ^∆∆^CT method. The primer sequences used were as follows: β-actin, Forward primer:5′-CATTGCTGACAGGATGCAGAAGG-3′ and Reverse primer:5′- TGC​TGG​AAG​GTG​GAC​AGT​GAG​G-3′; GLP-1R, Forward primer: 5′- CGG​AGT​GTG​AAG​AGT​CTA​AGC​G-3′ and Reverse primer:5′ -ATG​GCT​GAA​GCG​ATG​ACC​AAG​G -3′. Protein Expression Analysis: Min6 cells were treated with XMT for 48 h, and total protein was extracted. Western blot analysis was performed to detect GLP-1R protein levels, providing evidence of translational regulation.

### 2.6 Animal experiments

#### 2.6.1 Animal feeding and modeling

Healthy SPF male C57BL/6 mice (4 weeks old, 12–15 g) were purchased from Guizhou Huijiu Biotechnology Co., Ltd. and maintained under SPF conditions (light cycle: 12 h; humidity: 40%–50%; room temperature: 24°C ± 2°C; *ad libitum* access to sterile food and water). After 1 week of adaptive feeding, the mice were randomly divided into a normal group (n = 7) and a model group (n = 35). Mice in the model group were fed a high-fat high-sugar diet (HFD) providing 60% of energy from fat for 4 weeks. In the fifth week, they were intraperitoneally injected with freshly prepared 50 mg/kg STZ dissolved in citrate buffer (0.1 M, pH 4.5) for five consecutive days. Mice in the normal group were fed regular chow and injected with an equal volume of citrate buffer. Five days after the STZ injections, blood glucose levels were measured on two random days from tail vein samples. Mice with blood glucose levels ≥11.1 mmol/L were considered successful in the T2DM model.

#### 2.6.2 Grouping and management

Mice successfully modeled for T2DM were randomly assigned to five groups: model group, XMT low dose (0.655 g/kg/day), XMT medium dose (1.965 g/kg/day), XMT high dose (3.275 g/kg/day), and metformin group (250 mg/kg/day). Mice in the normal and model groups were given an equal volume of purified water. Body weight and fasting blood glucose levels were measured weekly for 8 weeks.

#### 2.6.3 Observation of T2DM mice symptoms

The water and food were weighed and supplemented per week, and the body weight of the mice was recorded. When changing the cages, food, water and bedding was weighed to evaluate the amount of water, food and urine of T2DM mice.

#### 2.6.4 Blood glucose and OGTT in mice

Fasting blood glucose levels of the mice were measured weekly at a fixed time. The night before the measurement, the mice were fasted but allowed free access to water. At week 8, an OGTT was conducted. After a 10-h fasting period, mice were orally administered 2 g/kg glucose, and blood glucose levels were measured at 0, 30, 60, 90, and 120 min using a glucose meter. The area under the curve (AUC) was calculated to compare glucose tolerance between the intervention and control groups.

#### 2.6.5 Biochemical indicators and insulin levels in mice

Serum HDL-C, LDL-C, TC, TG, GLU, AST, ALT, BUN, and CR levels were measured using kits (Nanjing Jiancheng, China). Serum insulin concentration was measured by ELISA (Jianglai Biology, China). Insulin resistance was estimated using the homeostasis model assessment of insulin resistance (HOMA-IR), calculated as: HOMA-IR = GLU × FINS/22.5.

#### 2.6.6 H&E staining of mice tissues

At the end of the experiment, pancreas tissue from mice was immediately isolated. A portion of the pancreas tissue from each group (n = 7) was fixed in 4% paraformaldehyde. After gradient dehydration with ethanol, the tissues were embedded in paraffin and sectioned using a pathological slicer (149AUTO00C1, Leica, Germany) (Shanghai Leica Instruments Co., Ltd. RM 2016). After H&E staining, slides were scanned using a Pannoramic 250 Flash III slide scanner (3DHistech, Ltd., Budapest, Hungary), and images were collected using CaseViewer software (3DHistech, Ltd., Budapest, Hungary).

#### 2.6.7 Immunofluorescence in mice

The slides were sequentially placed in deparaffinization solution, absolute ethanol, and distilled water. During antigen retrieval, care was taken to prevent excessive evaporation of the buffer, and the slides were not allowed to dry out. After natural cooling, the slides were washed three times in PBS (pH 7.4) using a shaking bed for 5 min each time. After drying slightly, 3% BSA was added for blocking for 30 min, followed by the addition of GLP-1R antibody (1:1000), Insulin antibody (1:500), and incubation overnight at 4°C. The slides were washed with PBS three times for 5 min each time, then incubated with CY3-labeled goat anti-mouse IgG and Alexa Fluor 488-labeled goat anti-rabbit IgG for 50 min in the dark at room temperature. After washing three times with PBS, DAPI was added and incubated in the dark at room temperature for 10 min. After washing three times with PBS, a fluorescence quenching agent was applied, and the slides were washed with water for 10 min. Finally, the slides were mounted with an anti-fluorescence quenching agent and observed under a fluorescence microscope to collect images.

### 2.7 Cell experiments

#### 2.7.1 Optimal time and concentration of XMT medicated serum in Min6 cells

Min6 cells were treated with XMT-containing serum at different volume fractions for 24, 48, and 72 h. MTT (10 μL/well) was added aseptically, and after 4 h, the supernatant was removed. MTT formazan crystals were dissolved in 160 μL DMSO, and the absorbance at 490 nm was measured using a microplate reader. Cell viability = (OD of the treatment group/OD of the control group) × 100%.

#### 2.7.2 Injury cell model and cell viability

Min6 cells were damaged using palmitic acid (PA) for 24 h to determine the damaging concentration. The Min6 cells were seeded at a concentration of 5 × 10^3^ cells/well in a 96-well plate and incubated overnight. Cells were then exposed to different concentrations of serum containing XMT and 0.15 mmol/L PA for 24 h. Groups included: Control group: treated with basal medium and 20% blank serum; Model group: treated with 0.15 mmol/L PA and 20% blank serum; 5% XMT group: treated with 0.15 mmol/L PA, 5% XMT-supplemented serum, and 15% blank serum; 10% XMT group: treated with 0.15 mmol/L PA, 10% XMT-supplemented serum, and 10% blank serum; 20% XMT group: treated with 0.15 mmol/L PA and 20% XMT-supplemented serum. All groups were treated with 500 nmol/L Liraglutide for 24 h, and cell viability was assessed using the MTT assay.

#### 2.7.3 Measurement Ca^2+^ concentration

Following the procedure described in 2.8.2, after drug treatment for a set period, the culture medium was removed, and Fluo-3 AM working solution (F8840, Solarbio) was added to the cells and incubated at 37°C for 30 min. The working solution was then removed, and the cells were washed three times with HEPES buffer. The cells were then incubated for 30 min with HBSS containing 1% BSA at 37°C. After ensuring complete de-esterification of AM inside the cells, fluorescence images were captured under a fluorescence microscope with excitation at 480–500 nm and emission at 525–530 nm.

#### 2.7.4 Glucose-stimulated insulin secretion (GSIS)

Min6 cells were seeded in a 96-well plate as described in 2.8.2. After three washes with Krebs-ringer bicarbonate HEPES buffer (KRBH), cells were incubated with KRBH for 1 h. Then, 2.8 mmol/L glucose in KRBH was added for 1 h. Afterward, the supernatant was collected. The cells were stimulated with 16.7 mmol/L glucose in KRBH for 1 h, and the supernatant was again collected and stored at −80°C. Finally, insulin levels were measured using a mouse INS ELISA kit (YJ302840, Jianglai Biology) according to the manufacturer’s instructions.

#### 2.7.5 Effect of inhibitor of GLP-1R in Min6 cell

Min6 cells were seeded in 6-well plates at a density of 30 × 10^4^ cells/well. After 24 h, the cells were treated with 20% XMT for 24 h. Following this, Exendin (9–39), a GLP-1R competitive antagonist, and Liraglutide were added, and the cells were incubated for an additional 24 h. The supernatants were then collected to measure cAMP secretion levels in all treatment groups.

### 2.8 Western blotting

Pancreatic tissues were immediately collected from the animals and homogenized using a tissue grinder. For the cell-based samples, Min6 cells were lysed using RIPA buffer (RIPA: PMSF = 100:1) for 1 h at 4°C. After centrifugation to remove cell debris, protein concentrations were determined using the BCA Protein Assay Kit (P0011, Beyotime). Protein samples were then separated by 10%–12% SDS-PAGE and transferred to PVDF membranes. The membranes were blocked with 5% skim milk in TBS for 1 h at room temperature. After blocking, the membranes were incubated overnight at 4°C with the following primary antibodies: Anti-β-actin (1:1000, 66009-1-Ig) Anti-GLP-1R (1:1000, YT 1916) Anti-PDX-1 (1:1000, 20989-1-AP). After washing with TBST, the membranes were incubated with the appropriate secondary antibody at room temperature for 2 h. The protein bands were visualized using the Odyssey infrared imaging system, and the intensities of the protein bands were analyzed with Image Studio software.

### 2.9 UHPLC-Q-TOF MS analysis

#### 2.9.1 Chromatographic conditions

Samples were analyzed using a SCIEX Exion LC Ultra High Performance Liquid Chromatography (UHPLC) system, equipped with a Phenomenex Kinetex^®^ C18 column (100 × 2.1 mm, 2.6 μm) and a Security Guard UHPLC C18 guard column for chromatographic separation. The column temperature was maintained at 40°C. The mobile phase consisted of 0.1% formic acid (v/v, Solvent A) and acetonitrile (Solvent B), with a flow rate of 0.4 mL/min. A 10 μL sample was injected into the system. The auto-sampler temperature was kept at 4°C throughout the analysis. The gradient elution program for chromatography is detailed in Table 1.

**TABLE 1:** Gradient elution flow chart.

Times (mins)	Ingredient gradients (B%)
0	1
1	1
10	100
13	100
14	1
17	1

#### 2.9.2 Q-TOF mass spectrometry analysis

The analysis was performed using a hybrid quadrupole time-of-flight mass spectrometer (Triple TOF™ 5600+; AB Sciex, Foster City, CA, USA) equipped with a Duospray ion source, in both positive/negative ESI ion modes for data collection. The ESI source conditions were set as follows: Nitrogen was used as both the nebulizer and auxiliary gas, with nebulizer gas 1 (GS1) at 55 psi; auxiliary gas (GS2) at 55 psi; curtain gas at 35 psi; ion source temperature set to 550°C; spray voltage of 5,500 V (+)/−4,500 V (−). In TOF MS-IDA-MS/MS data collection, the TOF MS spectral mass scan range was 100–1250 m/z, and the product ion scan mass range was 50–1,250 m/z. The TOF MS accumulation time was set to 0.10 s per spectrum, and the product ion scan accumulation time was set to 0.05 s per spectrum. Information-dependent acquisition (IDA) was used, with a high-sensitivity mode for product ion scanning. To improve the quality of the spectra, the following parameters for the IDA mode were applied: declustering voltage of 80 V (+)/−80 V (−), collision energy of 35 ± 15 eV (+)/−35 ± 15 (−), ion intensity not lower than 100 cps, excluding isotopes within 4 Da, and ion tolerance of 50 mDa. Each cycle monitored 10 candidate ions, and dynamic background subtraction (DBS) was enabled to enhance the sensitivity for low-abundance or trace analytes. An external calibration system (CDS) was used for automatic calibration of TOF MS and TOF MS/MS every four samples.

### 2.10 Statistical analysis

All data were expressed as mean ± standard deviation (SD). The significance of the variance analysis results was evaluated using SPSS 22.0 software. A P value <0.05 was considered statistically significant. Graphs were generated using GraphPad Prism 8.0 (GraphPad Software Inc., San Diego, CA, United States).

## 3 Results

### 3.1 The effect of XMT on GLP-1R expression

**FIGURE 1:**
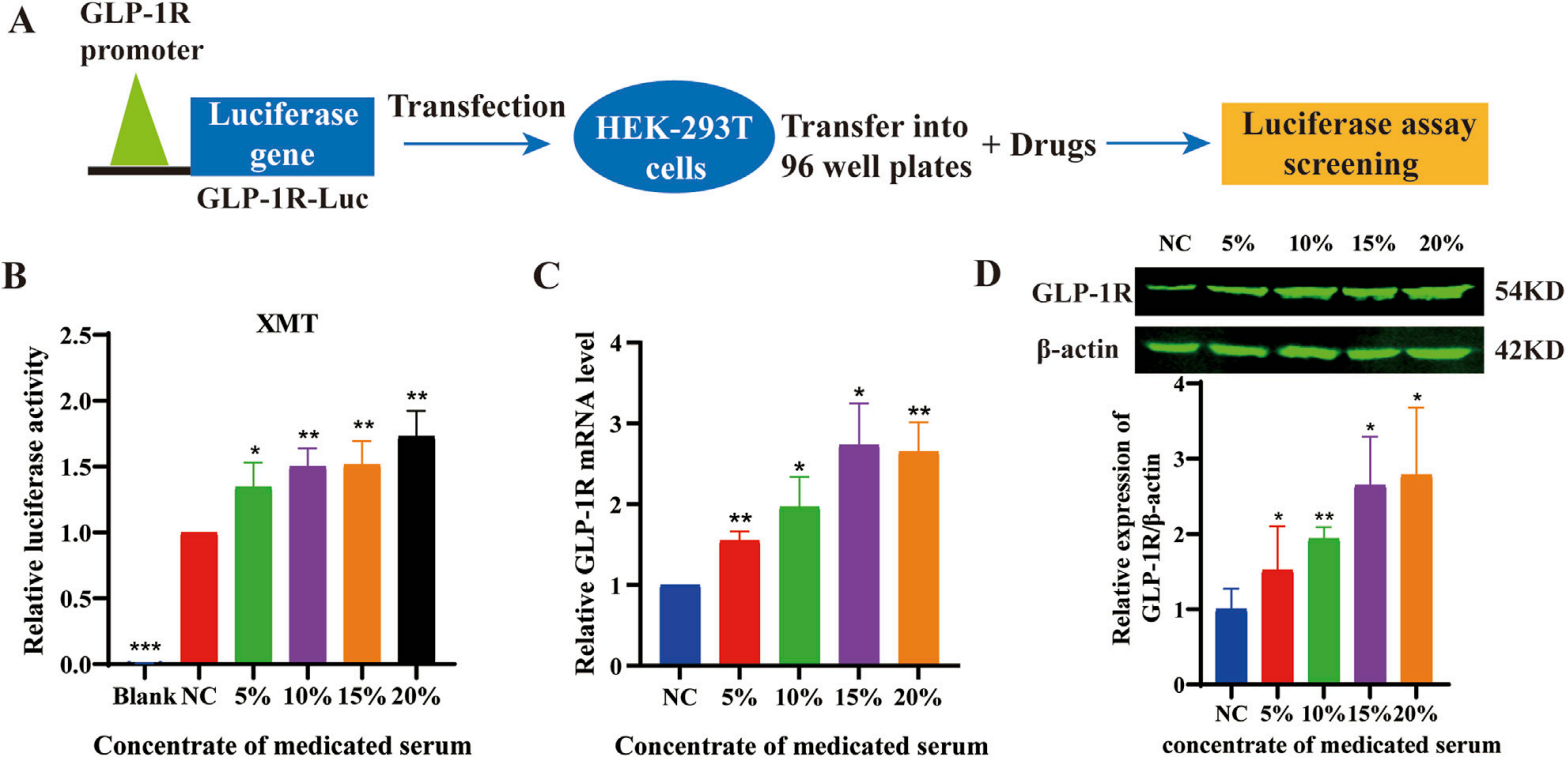
**(A)** Schematic diagram illustrating the drug screening process using the PGL3-GLP-1R-luc plasmid. **(B)** the luciferase activity in HEK-293T cells transfected with the PGL-3-GLP-1R-luc plasmid. **(C)** Analysis of GLP-1R mRNA levels in Min6 cells treated with different concentrations of XMT for 24 h **(D)** GLP-1R protein level in Min6 cells treated with different concentrations of XMT for 48 h. All data are expressed as the mean ± SD. Each experiment was repeated in triplicate. *P < 0.05, **P < 0.01, ***P < 0.001 vs. the NC group.

Using luciferase reporter constructs driven by GLP-1R promoter response elements (Figure 1A), We found that XMT significantly increased the luciferase intensity with dose-dependent manner in HEK-293T cells transfected with the PGL-3-GLP-1R-luc plasmid (Figure 1B). To further investigate whether XMT promote GLP-1R expression in pancreatic cells, we first measured the changes in GLP-1R mRNA levels using qPCR in Min6 cells treated with XMT. After 24 h of treatment, the transcription level of GLP-1R in Min6 cells was significantly elevated with dose-dependent manner (Figure 1C). Additionally, we assessed the protein expression of GLP-1R by Western blotting, which confirmed that XMT significantly increased GLP-1R protein level with dose-dependent manner in Min6 cells (Figure 1D).

### 3.2 Effects of XMT on body weight, urine, food intake, and water intake

**FIGURE 2:**
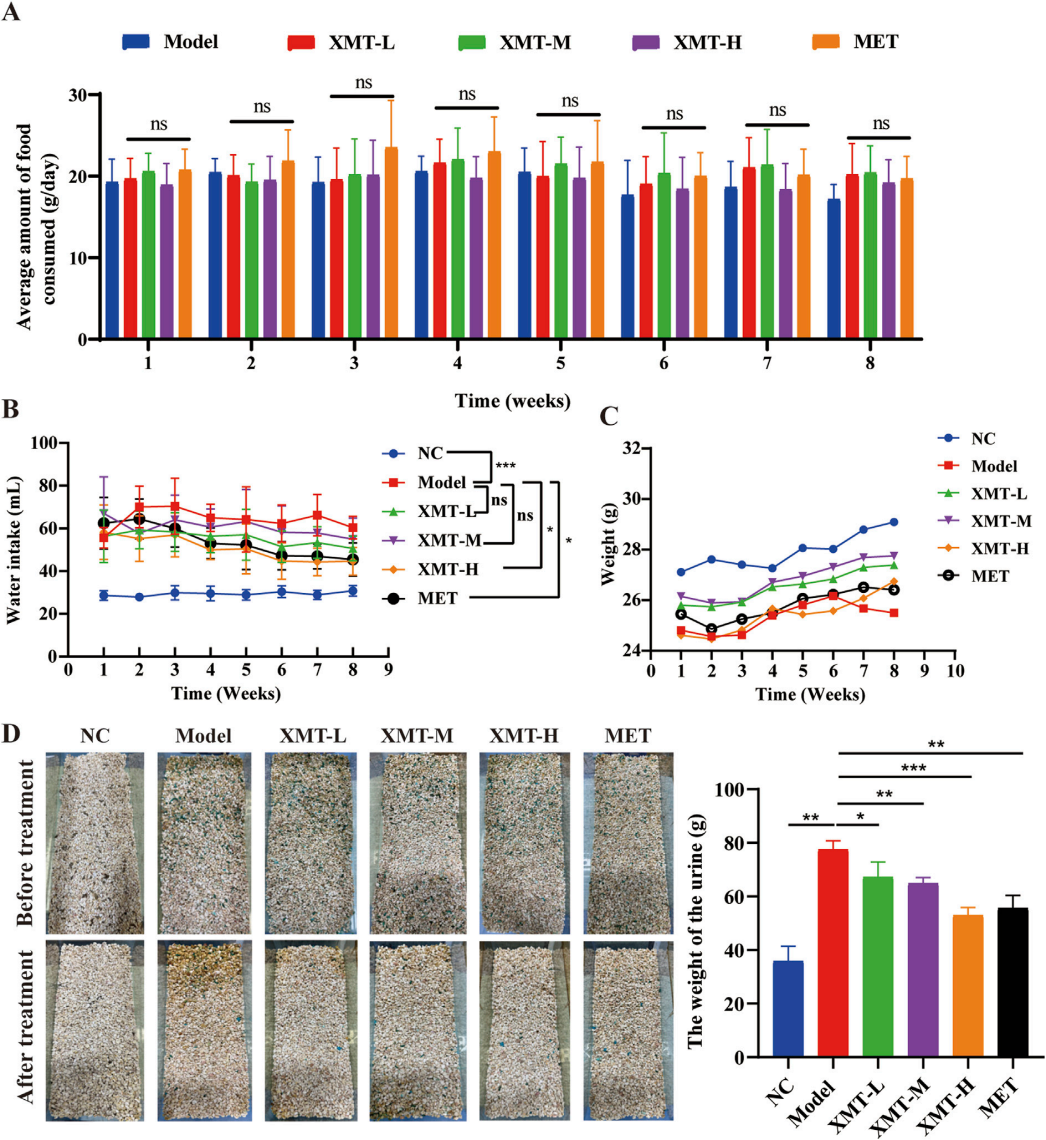
Effects of XMT on body weight, urine, food intake, and water intake. **(A)** The effects of XMT on Food intake **(B)** The effects of XMT on water intake **(C)** The effects of XMT on body weight. **(D)** The effects of XMT on Urine. All data are expressed as the mean ± SD. Each experiment was repeated in triplicate. Ns, no significant differences, *P < 0.05, **P < 0.01, ***P < 0.001 vs. the Model group.

Excessive drinking, eating, urine, and weight loss are the typical symptoms of T2DM. Therefore, food intake was monitored by recording the weight of the food added each time. The results showed that there were no significant differences in all groups (Figure 2A). Water intake was measured by recording the weight of water added. The results indicated that the amount of water intake was significantly lower in the groups treated with XMT or MET compared with the model group. Furthermore, the high-dose XMT or MET groups showed significant differences (Figure 2B). The model group mice experienced continued weight loss from the sixth week, while the XMT or MET treatment groups showed alleviation of this trend (Figure 2C). Urinary output was also recorded before and after treatment. The results showed that the amount of urine was significantly reduced in the XMT or MET groups compared with the model group (Figure 2D). These results indicated that the treatment of XMT can improve the “three more and one less” symptoms of T2DM mice, such as excessive drinking, urine, and weight loss.

### 3.3 The effects of XMT on blood glucose levels and insulin resistance in T2DM mice

**FIGURE 3:**
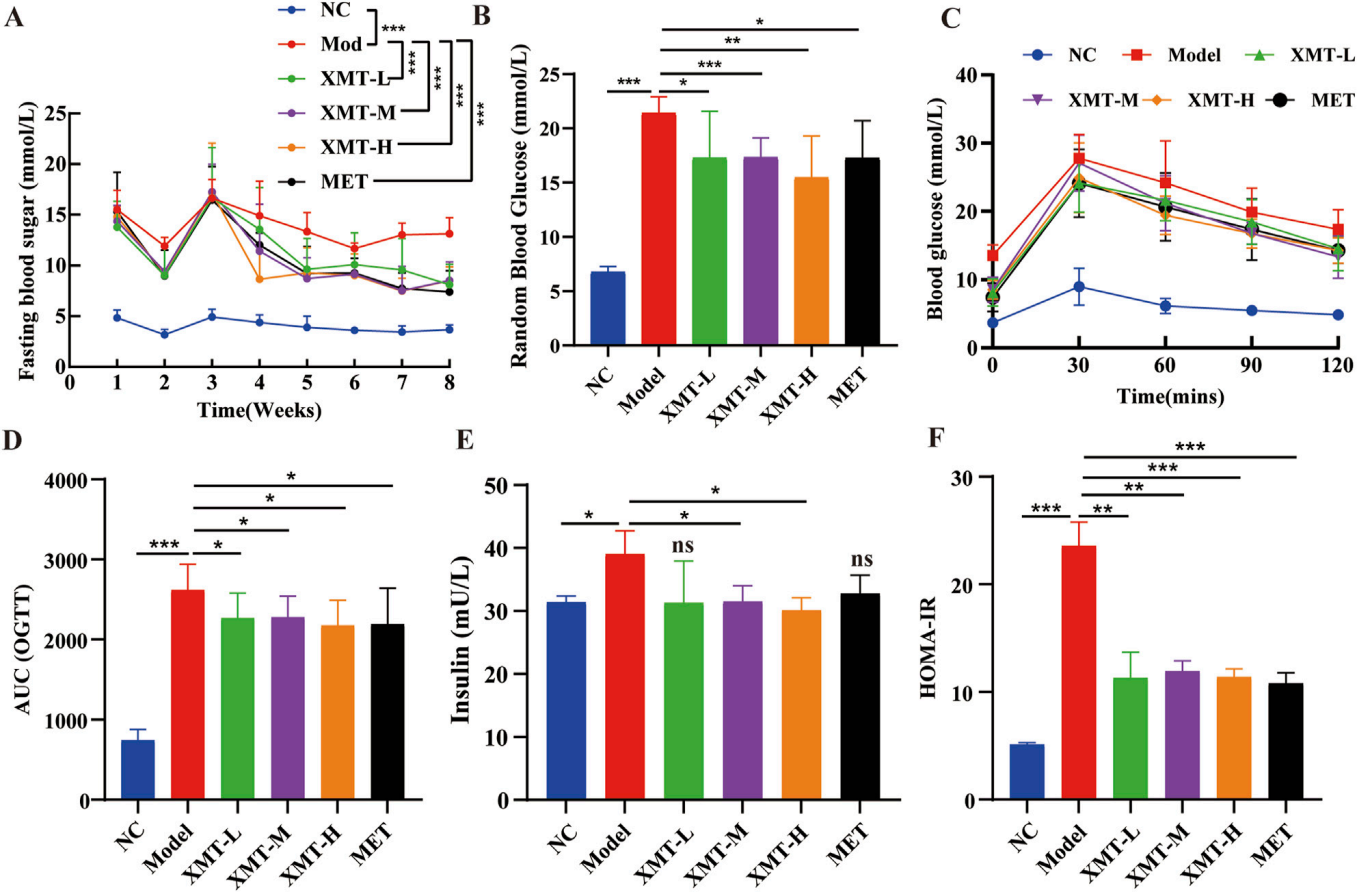
The effects of XMT on blood glucose levels and insulin resistance in T2DM mice. **(A)** Fasting blood glucose levels. **(B)** random blood glucose levels. **(C)** Blood glucose level tested by OGTT experiment. **(D)** AUC of OGTT. **(E)** Serum insulin level in mice. **(F)** HOMA-IR. All data are expressed as the mean ± SD. Each experiment was repeated in triplicate. Ns, no significant differences, *P < 0.05, **P < 0.01, ***P < 0.001 vs. the Model group.

To evaluate the effects of XMT on blood glucose levels, the T2DM mice were treated with XMT for 8 weeks. Then, the fasting blood glucose levels were measured once a week. Results showed that the fasting blood glucose levels in the model group were significantly higher than those in the normal control (NC) group. Following XMT treatment, the fasting blood glucose levels in the XMT-treated groups were significantly reduced (Figure 3A). To evaluate the effects of XMT on the postprandial blood glucose levels, Random blood glucose tests were conducted every 2 days at eighth week. The results showed that the groups treated with the XMT or MET showed a significant decrease in postprandial blood glucose levels compared with the control group (Figure 3B). OGTT results and the calculated AUC values indicated that both the XMT treatment groups and the MET group significantly improved glucose tolerance compared to the model group (Figures 3C, D). Furthermore, insulin levels and HOMA-IR values were significantly elevated in the model group compared to the NC group, indicating that T2DM mice have insulin resistance. After XMT treatment, insulin secretion was significantly reduced in XMT treatment groups compared to the model group. Moreover, the reductions in the medium or high-dose groups were statistically significant. Additionally, HOMA-IR scores were significantly lower in XMT treatment groups than in the model group (Figures 3E, F).

### 3.4 Effects of XMT on lipid metabolism, liver and kidney on T2DM mice

**FIGURE 4:**
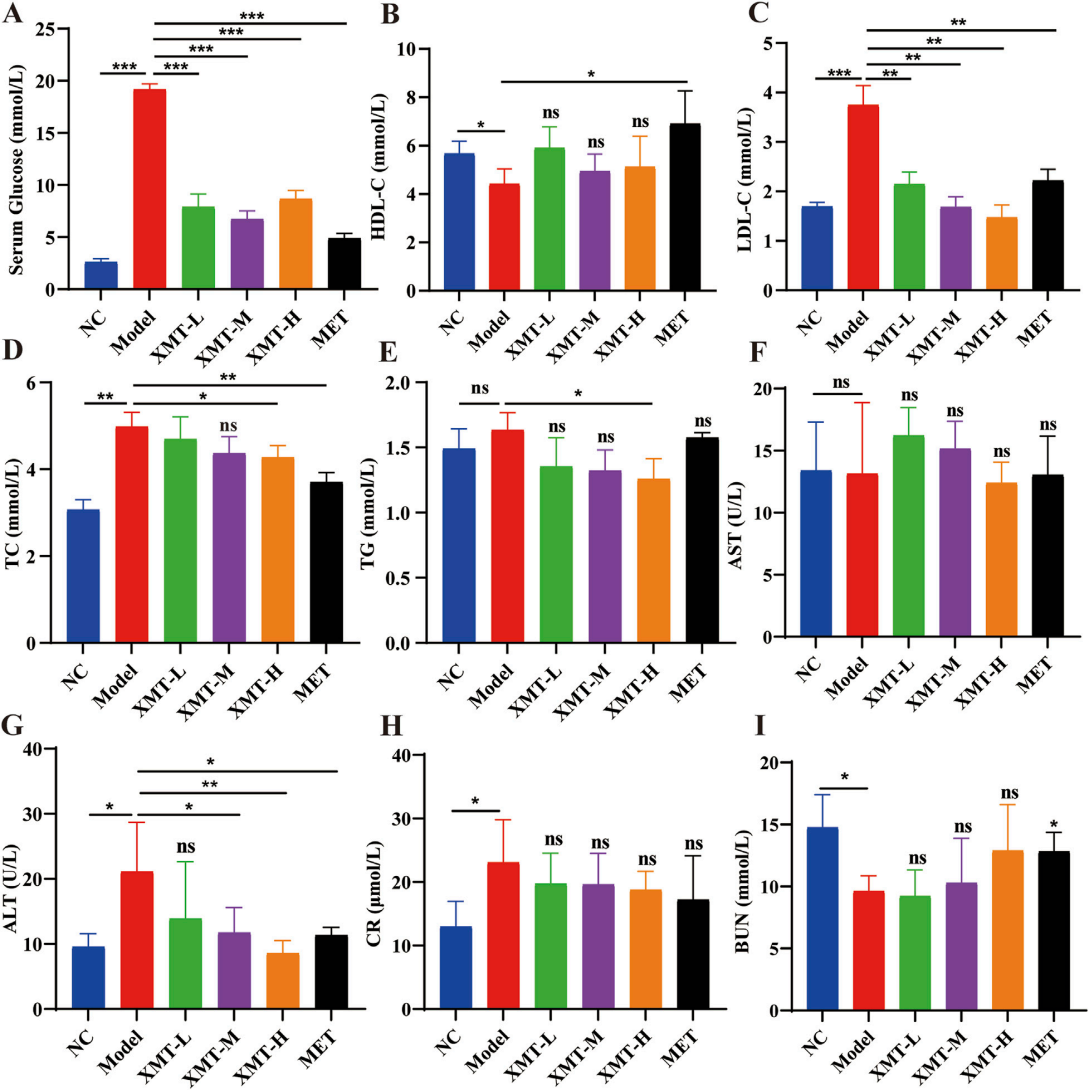
The effects of XMT on lipid metabolism, liver and kidney function in T2DM mice. **(A)** Glucose levels in the serum. **(B)** Serum HDL-C (high-density lipoprotein cholesterol) concentrations in mice. **(C)** Serum LDL-C (low-density lipoprotein cholesterol) concentrations in mice. **(D)** Serum TC (total cholesterol) concentrations in mice. **(E)** Serum TG (triglyceride) concentrations in mice. **(F)** Serum AST (Aspartate transaminase). **(G)** Serum ALT (Alanine aminotransferase). **(H)** Serum CR (Serum creatinine). **(I)** Serum BUN (Blood urea nitrogen). All data are expressed as the mean ± SD. Each experiment was repeated in triplicate. Ns, no significant differences, *P < 0.05, **P < 0.01, ***P < 0.001 vs. the Model group.

To further evaluate the effect of XMT on blood glucose levels in T2DM mice, the amount of glucose in their serum was detected using a Glucose kit. The results showed that the XMT treatment groups significantly reduced serum glucose levels (Figure 4A). At the same time, HDL-C levels were assessed using HDL-C kit. The results indicated that the HDL-C levels in model group were significantly lower than in the normal group. The XMT treatment groups increased HDL-C levels. However there is no significant difference compared with the model group (Figure 4B). LDL-C levels were evaluated using a LDL-C kit. The results showed that the XMT or MET treatment groups significantly reduced LDL-C levels compared with the model group (Figure 4C). TC levels were tested using a TC kit. The results showed that TC levels were significantly higher in the model group compared to the normal group. But both the XMT groups and the MET group reduced TC levels compared to the model group, with significant differences observed only in the XMT-H group or the MET group (Figure 4D). TG levels were assessed using a TG kit. The results showed that TG levels in the model group were slightly elevated compared to the normal group, but the difference was not statistically significant. After XMT treatment, TG levels were reduced, with significant differences observed only in the XMT-H group compared to the model group (Figure 4E). To evaluate the effect of XMT on liver function in mice, AST and SLT were tested using AST kits and ALT kits respectively. The results displayed that There was no significant difference in ALT between the treatment group and the model group. ALT in the model group was increased. The ALT level was reduced after XMT or MET treatment, and there were significant differences in groups treated with XMT-M, XMT-H or MET compared to the model group (Figures 4F, G). To evaluate the effect of XMT on kidney function in mice, CR and BUN were tested using CR kits and BUN kits respectively. The results showed the CR was higher in the model group compared to the NC group. The XMT or MET treatment group reduced CR. Although there is no significant difference compared with the model group. The model group had a significant reduction in BUN, while the XMT or MET groups reversed this trend. (Figures 4H, I). Above all, XMT effectively reduced serum glucose levels and improved lipid metabolism disorders in T2DM mice without affecting liver or kidney function.

### 3.5 Liver and pancreatic H&E staining in mice

**FIGURE 5:**
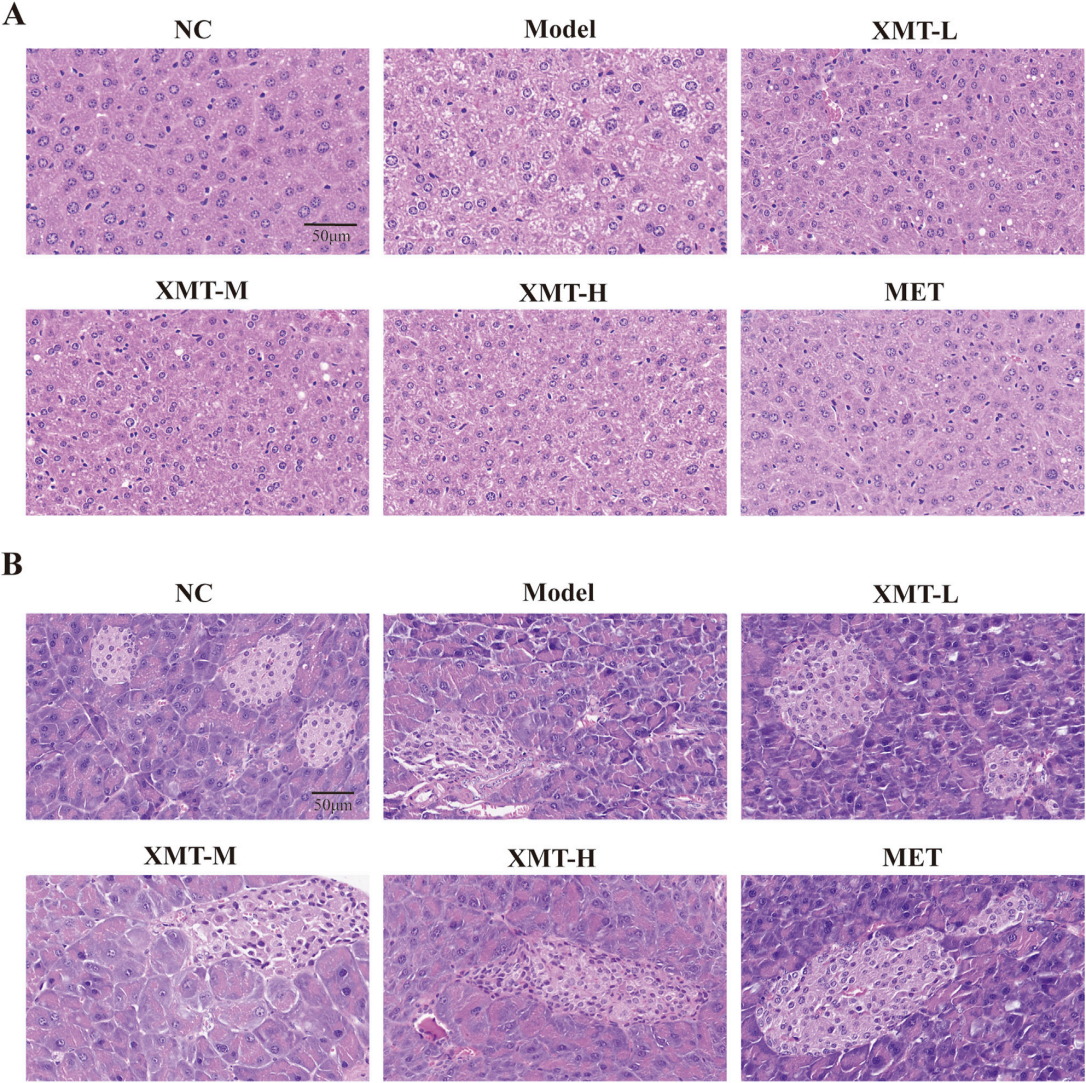
Liver and pancreatic H&E staining in mice. **(A)** Structural changes in liver tissues. **(B)** Structural alterations in pancreatic tissues.

To evaluate the effect of XMT on the liver tissue and pancreatic tissue of T2DM mice, we performed H&E staining. As shown in Figure 5A, H&E staining of liver tissues revealed significant pathological changes in the model group. Liver cells in the model group appeared visibly enlarged and exhibited pronounced macrovesicular steatosis (fatty degeneration). Treatment with XMT or MET partially ameliorated the pathological condition of the liver to varying extents. H&E staining of pancreatic tissues revealed that islet cells in the normal group were uniform and intact. In contrast, islet cells in the model group were deformed, and the structural integrity of the islet cell clusters was disrupted. Treatment with XMT and MET effectively improved these conditions and restored the structural integrity of the islet cell clusters (Figure 5B).

### 3.6 The effects of XMT reversing Min6 induced by PA injury

**FIGURE 6:**
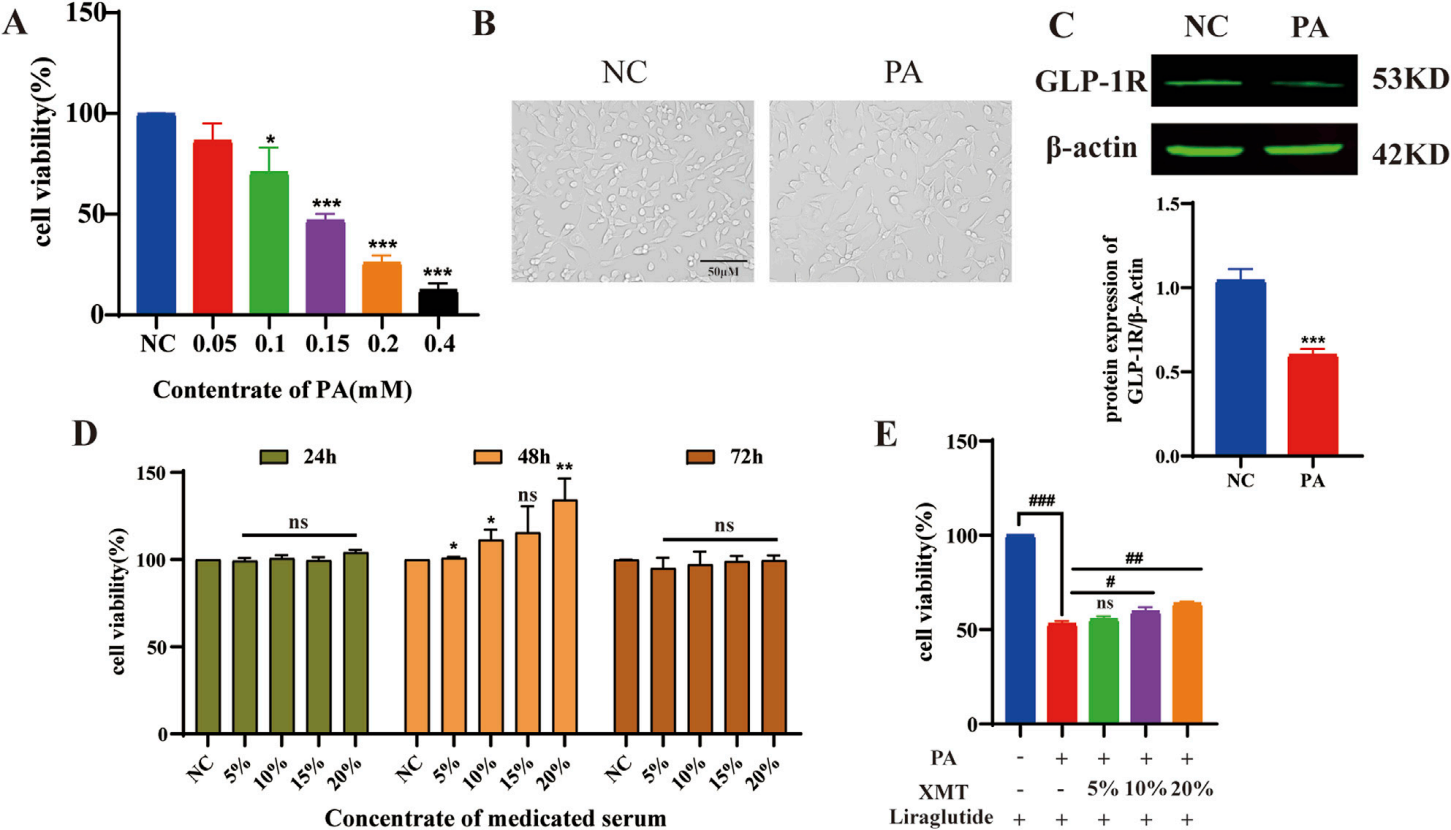
The effects of XMT on reversing injury Min6 induced by PA. **(A)** Screening of PA concentrations for the damage model in Min6 cells. **(B)** Microscopic images showing the morphology of PA-damaged Min6 cells. **(C)** GLP-1R protein expression and statistical analysis of GLP-1R levels after PA-induced damage in Min6 cells. **(D)** Determination of the optimal time and concentration for XMT treatment on Min6 cells. **(E)** XMT treatment restores the growth of PA-damaged Min6 cells. All data are expressed as the mean ± SD. Each experiment was repeated in triplicate. *P < 0.05, **P < 0.01, ***P < 0.001 vs. the NC group. ^#^ *P* < 0.05, ^##^ *P* < 0.01, ^###^ *P* < 0.001 vs. the PA group.

Min6 cells were exposed to varying concentrations of palmitic acid (PA) for 24 h, resulting in impaired cell growth. The 0.15 mM PA concentration was selected to establish the Min6 cell damage model (Figure 6A). PA inhibit the growth of Min6 cells (Figure 6B), and subsequent analysis of GLP-1R expression showed significant downregulation in PA-treated Min6 cells (Figure 6C). The optimal concentration and time for XMT treatment in Min6 cells were determined (Figure 6D). Notably, XMT treatment restored the growth of PA-damaged Min6 cells in a dose-dependent manner (Figure 6E).

### 3.7 Effects of XMT on the GLP-1R signaling pathway

**FIGURE 7:**
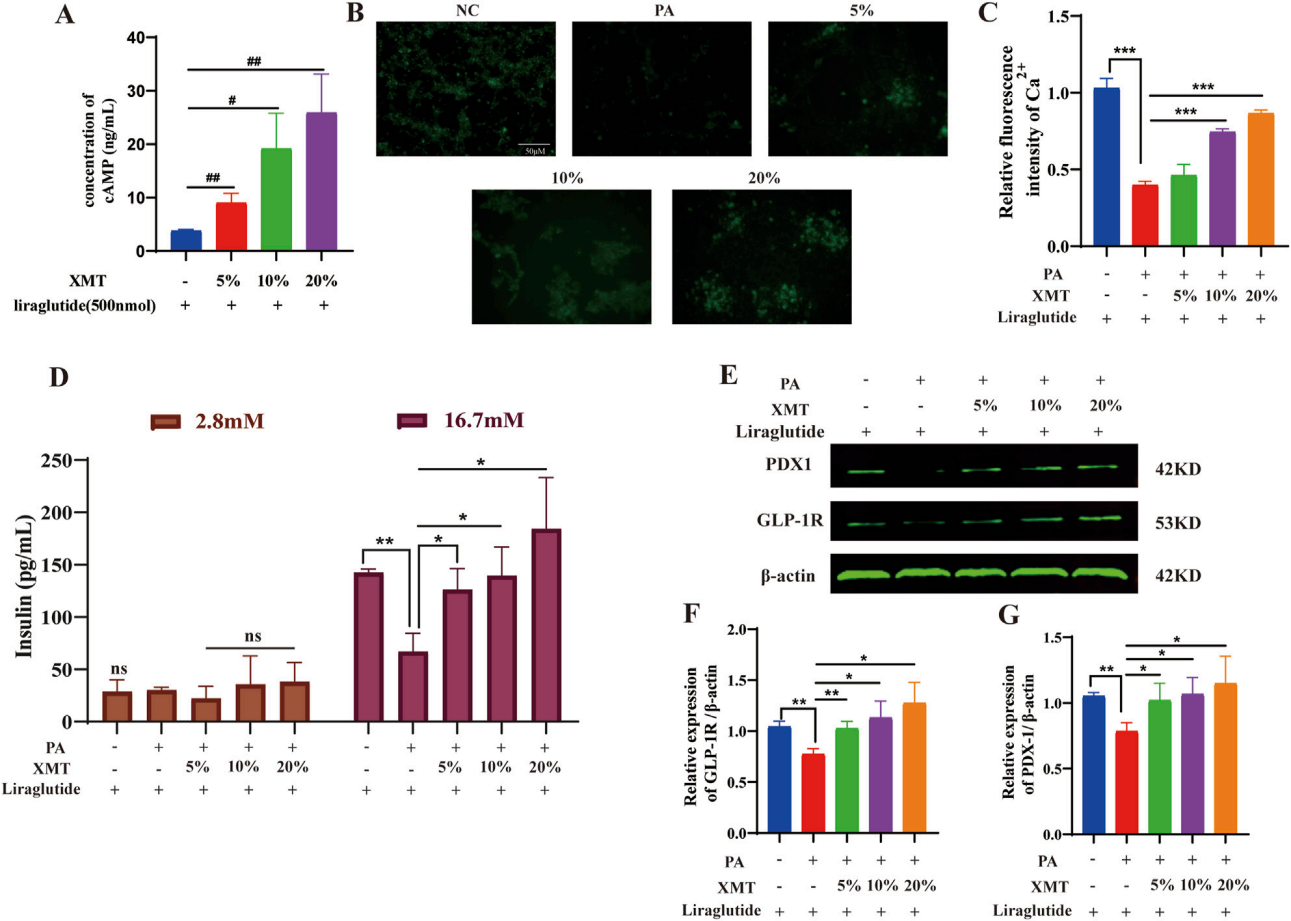
Effects of XMT on the GLP-1R signaling pathway. **(A)** Impact of XMT on cAMP release in Min6 cells. **(B)** Ca^2+^ fluorescence staining of PA-damaged Min6 cells treated with XMT. **(C)** Statistical analysis of Ca^2+^ fluorescence intensity. **(D)** Modulation of GSIS by XMT **(E)** Effects of XMT on GLP-1R signaling pathway-related proteins. **(F)** Statistical analysis of GLP-1R protein expression. **(G)** Statistical analysis of PDX-1 protein expression. All data are expressed as the mean ± SD. Each experiment was repeated in triplicate. ^#^ *P* < 0.05, ^##^ *P* < 0.01, ^###^ *P* < 0.001 vs. the NC group. NS, no significant differences. *P < 0.05, **P < 0.01, ***P < 0.001 vs. the PA group.

Previous findings indicated that XMT promotes GLP-1R protein expression. To further investigate its effects, Liraglutide, at the same concentration, was added to each group to measure cAMP release. The results demonstrated that cAMP release in the XMT groups was significantly higher than in the control group without XMT (Figure 7A). Additionally, we introduced XMT to the cell damage model and used a Ca^2+^ probe to assess Ca^2+^ influx. The results revealed that XMT significantly restored Ca^2+^ influx under cell damage conditions (Figures 7B, C). Furthermore, a GSIS experiment was conducted to evaluate the cellular response in different glucose environments, with insulin release being measured. The results showed that, under 16.7 mM glucose, insulin release was impaired in the PA group, whereas the XMT treatment restored this function (Figure 7D). Finally, Western blot analysis confirmed that XMT could restore the expression of GLP-1R and PDX-1 proteins, which were downregulated by PA-induced damage (Figures 7E–G).

### 3.8 The effect of GLP-1R inhibitor Exendin (9–39) on GLP-1R induced by XMT

**FIGURE 8:**
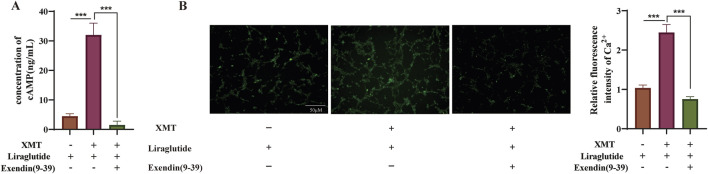
The effect of GLP-1R Inhibitor Exendin (9–39) on GLP-1R induced by XMT. **(A)** Effects of XMT on cAMP release in Min6 cells in the presence of Exendin (9–39). **(B)** Effects of XMT on Ca^2+^ influx in Min6 cells following Exendin (9–39) treatment. All data are expressed as the mean ± SD. Each experiment was repeated in triplicate. *P < 0.05, **P < 0.01, ***P < 0.001 vs. the XMT + Liraglutide group.

To assess whether Exendin (9–39) inhibits the GLP-1R expression induced by XMT, we measured cAMP release under conditions with or without Exendin (9–39). The results showed that Exendin (9–39) significantly reversed the release of cAMP (Figure 8A). Additionally, we evaluated the fluorescence intensity of Ca^2+^ and calculated the relative fluorescence intensity. The results demonstrated that the effect of XMT in promoting GLP-1R expression was inhibited, as evidenced by decreased cAMP release and suppressed Ca^2+^ influx (Figure 8B).

### 3.9 The effects of XMT on GLP-1R and insulin in pancreatic tissue by immunofluorescence

**FIGURE 9:**
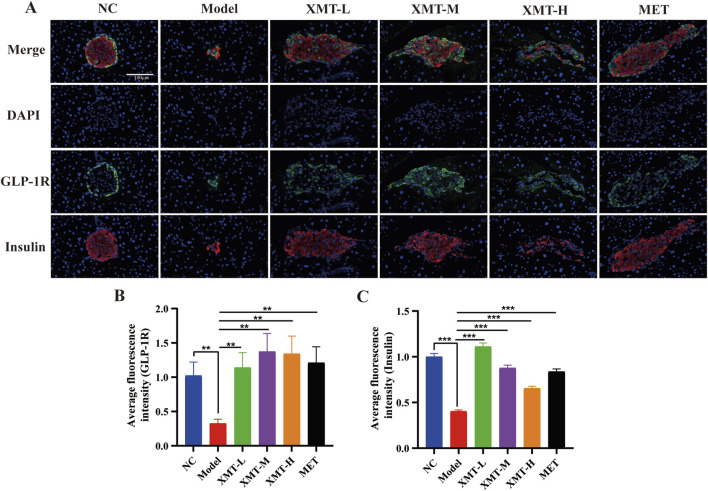
Immunofluorescence results. **(A)** Immunofluorescence staining of pancreatic tissues, GLP-1R (green), insulin (red) and DAPI staining (blue). **(B)** Statistical analysis of GLP-1R fluorescence expression. **(C)** Statistical analysis of insulin fluorescence expression. All data are expressed as the mean ± SD. Each experiment was repeated in triplicate. *P < 0.05, **P < 0.01, ***P < 0.001 vs. the Model group.

To assess the expression of GLP-1R and the release of insulin in pancreatic tissues, immunofluorescence experiments were conducted. The proteins GLP-1R and insulin were visualized using DAPI (blue), GLP-1R (green), and insulin (red) staining. The results indicated a significant reduction in GLP-1R protein or insulin protein in the model group. However, both the XMT and MET treatment groups demonstrated a notable restoration of GLP-1R protein or insulin protein compared to the model group (Figure 9A). There are significant differences compared to the model group (Figures 9B, C).

### 3.10 The effects of XMT on GLP-1R and PDX-1 in pancreatic tissue by western blots

**FIGURE 10:**
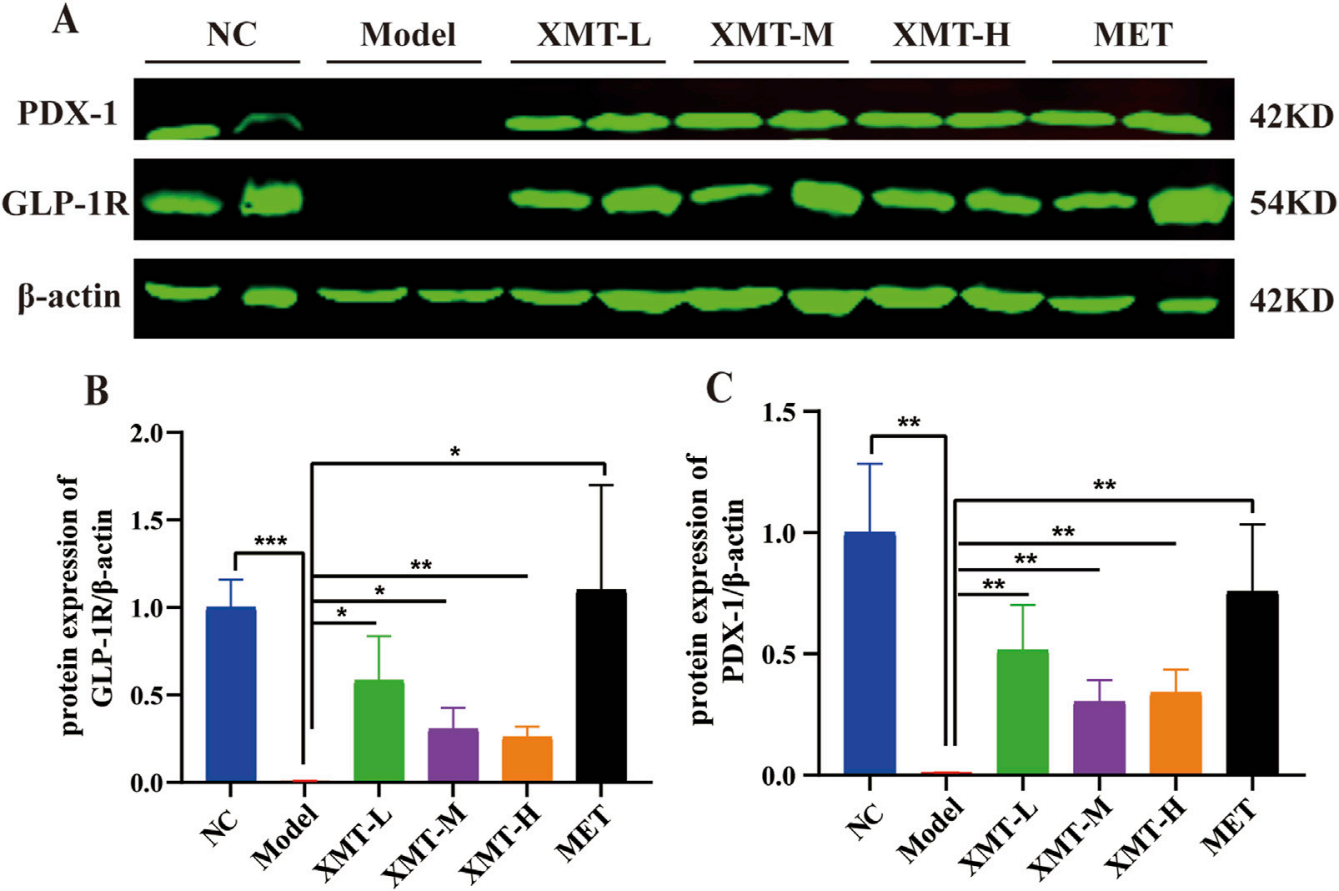
The effects of XMT on the changes of GLP-1R and PDX-1 in pancreatic tissue. **(A)** GLP-1R and PDX-1 protein in the pancreatic tissues of T2DM mice. **(B)** Statistical analysis of GLP-1R protein expression. **(C)** Statistical analysis of PDX-1 protein expression. All data are expressed as the mean ± SD. Each experiment was repeated in triplicate. *P < 0.05, **P < 0.01, ***P < 0.001 vs. the Model group.

Previous results indicated that XMT promotes the expression of GLP-1R. To further investigate the protective effects of XMT on pancreatic tissue and its underlying mechanisms for glucose-lowering, Western blotting was employed to assess the expression of GLP-1R and its downstream signaling protein, pancreatic duodenal homeobox-1 (PDX-1). As shown in Figure 10A, treatment with XMT or MET significantly increase the GLP-1R and PDX-1 protein levels in the pancreatic tissues of T2DM mice. There are significant differences compared to the model group (Figures 10B, C).

### 3.11 The analysis of the functional components in XMT by UHPLC-Q-TOF MS

**FIGURE 11:**
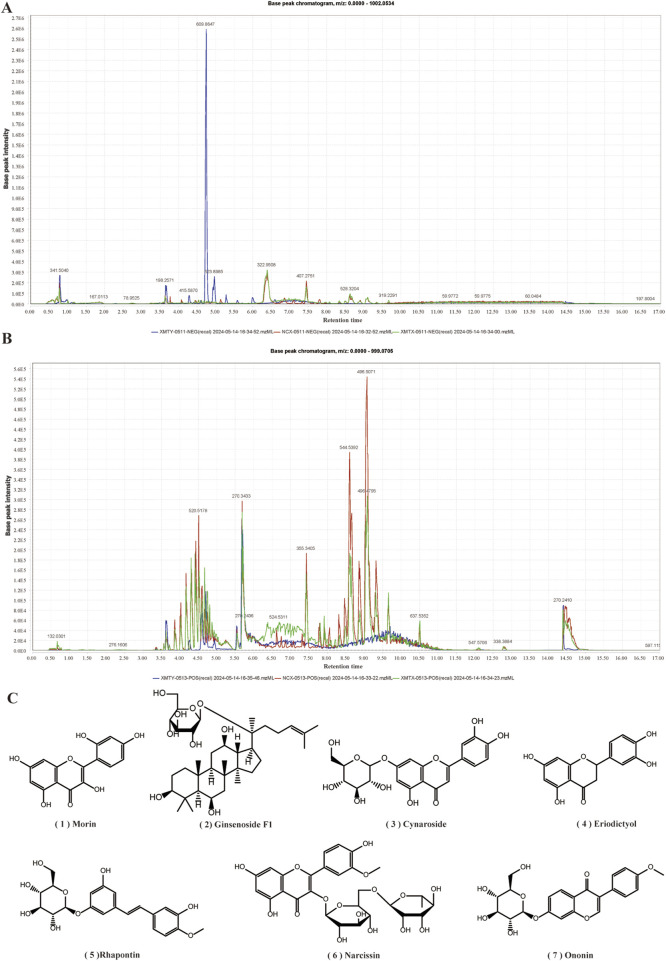
UHPLC-Q-TOF MS Analysis of Drug-Containing Serum from XMT. **(A)** Base peak chromatogram (BPC) under negative ion mode. **(B)** Base peak chromatogram (BPC) under positive ion mode. **(C)** Compounds from XMT in serum.

To assess the functional components in XMT, we analyzed the components in three groups of samples: the original ingredient of XMT, blank group serum, and drug-containing serum from XMT. The results revealed the presence of seven compounds from the XMT in the drug-containing serum, identified as: morin, ginsenoside F1, cynaroside, eriodictyol, rhapontin, narcissoside and ononin (Figure 11).

### 3.12 Effects of XMT serum compounds on GLP-1R protein level and release of cAMP an insulin in Min6 cells

**FIGURE 12:**
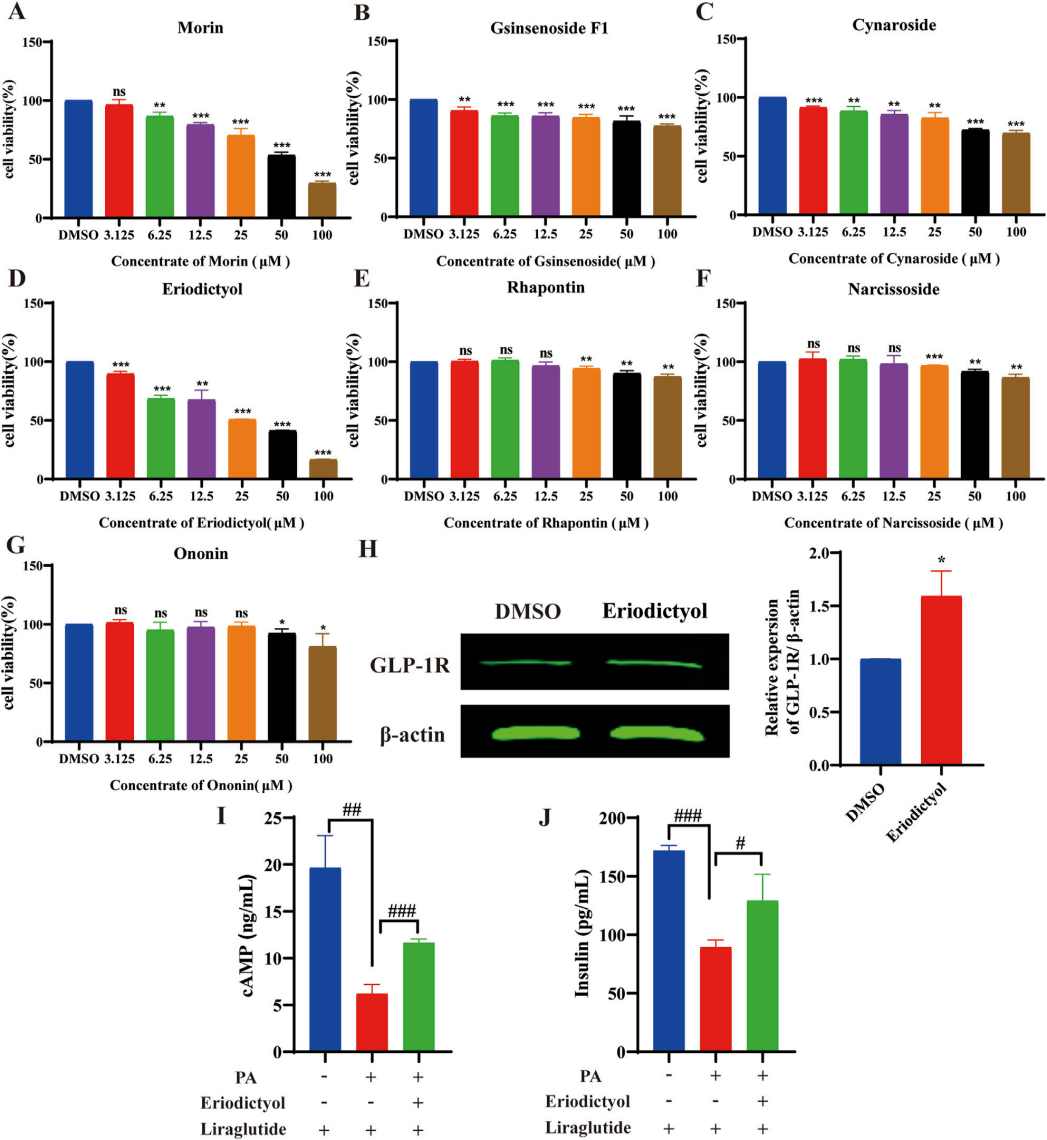
The results of activity screening in the XMT serum component. **(A)** The active screening results of morin **(B)** The active screening results of ginsenoside F1, **(C)** The active screening results of cynaroside. **(D)** The active screening results of eriodictyol. **(E)** The active screening results of rhapontin. **(F)** The active screening results of narcissoside. **(G)** The active screening results of Ononin. **(H)** The effect of eriodictyol on GLP-1R protein expression. **(I)** Effects of eriodictyol on cAMP release in Min6 cells. **(J)** Effects of eriodictyol on insulin release in Min6 cells. All data are expressed as the mean ± SD. Each experiment was repeated in triplicate. *P < 0.05, **P < 0.01, ***P < 0.001 vs. the DMSO group. ^#^ *P* < 0.05, ^##^ *P* < 0.01, ^###^ *P* < 0.001 vs. the PA + Liraglutide group.

To identify the concentration of compounds from XMT serum that can increase GLP-1R protein levels without inducing toxicity, we screened seven compounds using the MTT assay. The results indicated that the compounds morin, ginsenoside F1, cynaroside and eriodictyol exhibited no toxicity to Min6 cells at a concentration of 3.125 µM (Figures 12A–D). Rhapontin, narcissoside and Ononin showed no toxicity to Min6 cells at a concentration of 50 μM, 50µM, 25 µM respectively (Figures 12E–G). Notably, eriodictyol was found to significantly upregulate the expression of GLP-1R and promote the release of cAMP and insulin. (Figures 12H–J).

## 4 Discussion

XMT has long been used in clinical treatments for hypertension, hyperlipidemia, and other conditions, guided by the principles of TCM. Our research shows that XMT has a significant hypoglycemic effect, improves glucose tolerance in T2DM mice, and enhances insulin sensitivity. However, XMT did not reduce the excessive food intake observed in T2DM mice, despite consistent dietary conditions between the model and XMT treatment groups. Additionally, weight loss was observed in the high-dose group during the first 6 weeks, but this phenomenon improved significantly after week six. These observations could be partly due to individual differences among animals, and there may be unidentified negative factors affecting body weight in the high-dose group, which warrants further investigation.

In addition to elevated blood glucose levels, T2DM patients typically experience dyslipidemia, characterized by increased levels of triglycerides (TG), total cholesterol (TC), low-density lipoprotein cholesterol (LDL-C), and decreased high-density lipoprotein cholesterol (HDL-C). A previous meta-analysis suggested that combining XMT with conventional Western medicine is more effective than Western medicine alone in reducing TG, TC, and LDL-C levels (Ji et al., 2022). Our results indicate that XMT treatment improves lipid metabolism in T2DM mice by reducing TG, TC, and LDL-C levels. In particular, all XMT treatment groups significantly improved LDL-C levels, with the high-dose group (XMT-H) showing greater efficacy compared to the low- and medium-dose groups (XMT-L and XMT-M). However, only the high-dose group significantly reduced TC and TG levels. Notably, we did not observe significant improvements in HDL-C levels, consistent with previous reports that XMT combined with conventional western medicine treatment did not improve HDL-C more significantly. This outcome was anticipated. Mechanistic analyses imply that XMT may not affect HDL-C synthesis, metabolism or reverse cholesterol transport. After the blood glucose is reduced, XMT causes a slight increase of HDL-C through other lipid metabolism pathways (such as observed LDL-C or TG reduction). Nevertheless, XMT treatment can help prevent the continuous decrease in HDL-C levels. A recent study suggested that excessively high HDL-C levels might also increase the risk of cardiovascular diseases (Perswani et al., 2024), highlighting the need for further comprehensive research on XMT’s effects on HDL-C. However, this study does not investigate the effect of XMT combining with conventional Western medicine on T2DM. In future, we will study the effect of XMT combined with GLP-1R agonist on T2DM.

Insulin resistance, a common feature of obesity and T2DM, is associated with elevated concentrations of long-chain free fatty acids (LC-FFA) in the blood (Wrzosek et al., 2022). These elevated LC-FFAs are believed to contribute to pancreatic β-cell death, a fundamental feature of T2DM development (Thomas et al., 2023; Mastrototaro and Roden, 2021). As β-cell damage increases, their ability to release insulin and respond to insulin sensitizers is also compromised (Lin et al., 2023). Our study demonstrates that XMT exerts hypoglycemic effects in T2DM mice by promoting GLP-1R expression in β-cells and improving the microstructure of pancreatic and liver cells. In conclusion, our results support the significant anti-T2DM effects of XMT.

To explore the effects of XMT on Min6 cells and its potential mechanisms, we established a palmitic acid (PA)-induced Min6 cell injury model (Choi et al., 2023; Lee et al., 2022). After 24 h of exposure to PA, we observed inhibited growth and significant damage to GLP-1R protein expression in Min6 cells. However, pre-treatment with serum containing XMT significantly alleviated these harmful effects, suppressing cell death and promoting cell growth. Although the growth rate did not improve rapidly, the effects remained significant. These results suggest that XMT-containing serum can significantly prevent damage caused by elevated PA levels in Min6 cells.

Under normal physiological conditions, GLP-1R is activated by GLP-1, which stimulates adenylate cyclase (AC), initiating the production of cAMP (Müller et al., 2019). Our study demonstrated that supplementing Min6 cells with serum containing XMT, and further adding Liraglutide, increased the cAMP concentration in a dose-dependent manner. This suggests that XMT can promote GLP-1R expression in Min6 cells. After activation by Liraglutide, the release of cAMP followed the same trend. Previous studies have shown that prolonged exposure to high levels of long-chain fatty acids can damage GLP-1R expression (Wu et al., 2023). To further validate XMT’s promotion of GLP-1R, we used the PA injury cell model. Our results show that the Ca^2+^ concentration in the PA group was significantly reduced, while supplementation with XMT serum restored this decrease in Ca^2+^ concentration. Changes in Ca^2+^ concentration trigger insulin release from pancreatic β-cells, which is mediated by exocytosis in response to increased local Ca^2+^ levels in insulin secretory granules (Rorsman and Ashcroft, 2018). Accordingly, we also measured insulin secretion levels and observed that XMT restored insulin secretion in PA-damaged cells under 16.7 mM glucose stimulation.

Pancreatic and duodenal homeobox 1 (PDX-1) plays a crucial role in pancreatic development and maintaining β-cell function (Zhang et al., 2020). Studies have shown that GLP-1 regulates PDX-1, promoting its expression in a glucose-dependent manner, increasing intracellular protein levels, and enhancing binding to the insulin gene promoter (Zhang et al., 2022). Our results show that XMT supplementation significantly increased the expression of both GLP-1R and PDX-1 in PA-exposed Min6 cells, highlighting XMT’s potential to restore GLP-1R and PDX-1 activity. XMT promotes GLP-1R expression, enhances Ca^2+^ concentration in pancreatic β-cells, and activates insulin release, thereby restoring PDX-1 protein activity in Min6 cells.

Previous research on GLP-1R has identified compounds such as Morus alba polysaccharides and Berberine, which promote GLP-1 secretion through different pathways (Xie et al., 2020; Yang et al., 2024), offering insights for developing treatments for T2DM. There has also been extensive research on GLP-1R agonists, including newly synthesized peptide compounds and plant-derived small molecules (Shang et al., 2023; Zimmermann et al., 2022). These findings have informed our current work. Although we previously investigated whether XMT could promote GLP-1 secretion from intestinal L cells, the results were unsatisfactory. We also explored whether XMT could function as a GLP-1R agonist. Our experimental results showed no difference in cAMP concentration in Min6 cells with XMT alone, without a GLP-1R agonist. Ultimately, we found that XMT promotes GLP-1R expression and enhances the sensitivity of GLP-1R to GLP-1. This is different from the traditional drugs that mainly stimulate the secretion of insulin by islet β cells, the role of GLP-1R is glucose-dependent. In addition, compared with modern western medicine, such as GLP-1R agonists have the risk of nausea, diarrhea, vomiting, constipation and pancreatitis (Liu et al., 2022), while XMT has the characteristics of multi-component, multi-target and less adverse reactions.

Currently, the chemical composition of XMT remains unclear. LC-MS analysis identified seven blood components from XMT, including morin, ginsenoside F1, cynaroside, eriodictyol, rhapontin, narcissoside and ononin. Morin has a certain effect on improving insulin resistance and lipid accumulation in pre-diabetic mice, and improving insulin resistance by activating PPAR γ (Ren et al., 2023). Ginsenoside F1 can play an anti-cancer role by regulating insulin-like growth factor 1 (IGF-1). Although this report has not been applied in T2DM, IGF-1 is closely related to insulin (Kwon et al., 2018). Cynaroside has been reported to have enhancing and anti-T2DM effects (Bouyahya et al., 2023). Eriodictyol can stimulate insulin secretion by regulating the cAMP/PKA signaling pathway in mouse islets (Hameed et al., 2018). It was also reported that different concentrations of insulin were added to 3T3-L1 cells, and then rhaponticin was added to the cells to observe the glucose uptake of the cells. The results showed that rhaponticin could significantly promote the glucose uptake of the cells (Choi et al., 2006). Although there are few reports on narcissoside, a molecular docking simulation determined the interaction between DPP-4 and narcissoside, and inserted the active site cavity of DPP-4 and interacted with the key amino acid residues at the active site (Pan et al., 2022). Ononin has been reported to inhibit early adipogenic differentiation, which also has a certain effect on the prevention of T2DM (Mladenova et al., 2022). Among them, eriodictyol can improve dyslipidemia, fatty liver and IR in diet-induced obese mice (Kwon and Choi, 2019), promote insulin release through the cAMP/PKA pathway, which is related to the activation of GLP-1R. At the same time, we obtained the result that eriodictyol can promote the expression of GLP-1R in Min6 cells, which indicates that eriodictyol is worthy of further exploration in the study of T2DM. These compounds constitute the blood components of XMT, which proves that XMT has the effect of treating T2DM. Moreover, these compounds may undergo metabolism to form new metabolites in the body, a subject that could be explored in future pharmacokinetic and compound analysis studies to better understand the mechanisms of XMT in T2DM treatment.

## 5 Conclusion

In conclusion, our study demonstrates that XMT has significant hypoglycemic effects in T2DM mice, providing substantial protection to the pancreas. This protective effect is likely attributed to XMT’s ability to promote the expression of GLP-1R, enhancing pancreatic function. However, several limitations exist in this study. Notably, in the *in vivo* experiments, the high, medium, and low doses of XMT did not show a dose-dependent effect on certain indicators, raising concerns about potential unknown side effects from long-term high-dose XMT intake. Despite this, our findings indicate that high-dose XMT does not adversely affect liver or kidney function, suggesting that it may be safe for short-term use. Additional research is necessary to confirm these results and establish the optimal dosage for therapeutic use.

Moreover, in the *in vitro* experiments, we used serum containing XMT-derived components. We conducted an analysis of XMT’s blood components using Ultra high-performance liquid chromatography-mass spectrometry (UHPLC-MS) and identified seven compounds. At the same time, they were screened for viability and found that Eriodictyol could increase the expression of GLP-1R However, a more in-depth analysis of these chemical components was not performed. Future studies should aim to explore the individual components of XMT in serum and further investigate their specific roles in promoting GLP-1R expression. Additionally, further research is required to clarify the exact mechanisms through which XMT enhances GLP-1R expression and its broader implications for T2DM treatment.

## Data Availability

The original contributions presented in the study are included in the article/supplementary material, further inquiries can be directed to the corresponding author.
